# Microbial Fabrication of Nanomaterial and Its Role in Disintegration of Exopolymeric Matrices of Biofilm

**DOI:** 10.3389/fchem.2021.690590

**Published:** 2021-05-24

**Authors:** Moupriya Nag, Dibyajit Lahiri, Tanmay Sarkar, Sujay Ghosh, Ankita Dey, Hisham Atan Edinur, Siddhartha Pati, Rina Rani Ray

**Affiliations:** ^1^ Department of Biotechnology, University of Engineering and Management, Kolkata, India; ^2^ Department of Food Technology and Bio-Chemical Engineering, Jadavpur University, Kolkata, India; ^3^ Malda Polytechnic, West Bengal State Council of Technical Education, Government of West Bengal, Malda, India; ^4^ AMH Energy Pvt. Ltd., Kolkata, India; ^5^ Department of Biotechnology, Maulana Abul Kalam Azad University of Technology, Haringhata, India; ^6^ School of Health Sciences, University Sains Malaysia, Kelantan, Malaysia; ^7^ Centre of Excellence, Khallikote University, Berhampur, India; ^8^ Research Division, Association for Biodiversity Conservation and Research (ABC), Balasore, India

**Keywords:** microbial nanomaterials, antibiofilm, exopolysaccharide, medical devices, nanotechnology, bioprospecting

## Abstract

Bacterial biofilms are responsible for the development of various chronic wound-related and implant-mediated infections and confer protection to the pathogenic bacteria against antimicrobial drugs and host immune responses. Hence, biofilm-mediated chronic infections have created a tremendous burden upon healthcare systems worldwide. The development of biofilms upon the surface of medical implants has resulted in the failure of various implant-based surgeries and therapies. Although different conventional chemical and physical agents are used as antimicrobials, they fail to kill the sessile forms of bacterial pathogens due to the resistance exerted by the exopolysaccharide (EPS) matrices of the biofilm. One of the major techniques used in addressing such a problem is to directly check the biofilm formation by the use of novel antibiofilm materials, local drug delivery, and device-associated surface modifications, but the success of these techniques is still limited. The immense expansion in the field of nanoscience and nanotechnology has resulted in the development of novel nanomaterials as biocidal agents that can be either easily integrated within biomaterials to prevent the colonization of microbial cells or directly approach the pathogen overcoming the biofilm matrix. The antibiofilm efficacies of these nanomaterials are accomplished by the generation of oxidative stresses and through alterations of the genetic expressions. Microorganism-assisted synthesis of nanomaterials paved the path to success in such therapeutic approaches and is found to be more acceptable for its “greener” approach. Metallic nanoparticles functionalized with microbial enzymes, silver–platinum nanohybrids (AgPtNHs), bacterial nanowires, superparamagnetic iron oxide (Fe_3_O_4_), and nanoparticles synthesized by both magnetotactic and non-magnetotactic bacteria showed are some of the examples of such agents used to attack the EPS.

## Introduction

Global mortality and morbidity is maximally associated with infectious diseases and is one of the profound causes for the development of antibiotic resistance. It has been observed that after 1980, pharmaceutical companies stopped manufacturing novel antibiotics due to the lack of returns with respect to the investment, high cost associated with the development of drugs, and prolonged time requirement and for the rapid development of resistances (Whitchurch et al., 2002). The development of phenotypic resistances results in the amplification of resistances associated with genes toward diverse types of disinfectants and antibiotics. Biofilms are the group of organized colonies of microbial species comprising fungi, bacteria, and yeasts that develop a syntrophic association with their adherence to the biotic and abiotic surfaces by self-encapsulating extracellular polymeric substances (EPSs) (Costerton et al., 1995). The microcolonies existing within the EPS interact via the mechanism of quorum sensing (QS) that specifically helps in the development of the biofilm and the expression of virulence (Pircalabioru and Chifiriuc, 2020). The phenotypic and genotypic expressions of the sessile cells differ from the planktonic forms and are majorly associated with the development of resistances against antibiotics. Antibiotic resistance is actually imparted by the EPS, which prevents the penetration of the antibiotics and also induces the multidrug efflux pumps within the biofilm and thus results in the development of persister cells (Mah and O’Toole, 2001; Lewis, 2005). The metastasis of the sessile cells from the mature biofilm results in the transmission and dissemination of biofilm-associated infections (Nikolaev and Plakunov, 2007; Dongari-Bagtzoglou, 2008). The various infections that are associated with the biofilm on various biomedical surfaces are considered to be dangerous in healthcare sectors in comparison to the planktonic forms (Allegranzi et al., 2011; Zarb et al., 2012).This has resulted in the urgency to develop alternate therapeutic strategies to combat biofilm-associated infections, precisely through disintegration of the EPS matrix.

The field of nanotechnology involves scientific and engineering technologies that aim to synthesize various materials of nano-dimensions that have wide applications in the fields of bioprospecting, pharmaceuticals, human activities, and biomedical applications. The development of nanomaterials is a new and promising strategy for acting as therapeutic agents against various types of biofilm-associated pathogenic infections that are associated with implants and medical devices (Pircalabioru and Chifiriuc, 2020). Various types of nanomaterials have been associated to combat against various biofilms due to their prevailing properties which are microbiostatic, microbiocidal, and antipathogenic in nature and because they can be used for the purpose of delivering synthetic drugs and natural compounds (Grumezescu and Chifiriuc, 2014). Most of the nanoparticles (NPs) are metallic in nature and comprise metallic oxides, metal-based polymeric composites, polymers, chitosan-based nanomaterials, peptides, combinations of nanoparticle-associated antibiotics, and nanomaterials which have efficacy of antimicrobial agents without bringing about any damage to the host (Pati et al., 2020; Pati et al., 2021).

Although a number of reports are available on the antibiofilm activities of nanomaterials, like carbon nanotubes (Kang et al., 2007), oxygen-deficient zinc oxide (ZnO) nanowires (Elbourne et al., 2020), and core–shell nanofiber membranes loaded with silver nanoparticles (Alharbi et al., 2018), a very scanty number of reports are available on the nanomaterials formed from a microbial source. Since application of microbiogenic nanomaterials that can be used for the disruption of the biofilm matrix may be a significant strategy to combat biofilm-mediated infections, the present study presents an overview of nanomaterials synthesized from various microbial sources, their characteristic features, and their antibiofilm nature with a critical elucidation of their mode of action.

## Microbial Synthesis of Nanomaterials

Nanomaterials, with dimensions lower than 100 nm, have attracted the interest of scientists due to their quantum size effect with the variation of electronic properties. Nanomaterials can have one, two, or three dimensions in the nanoscale, as exemplified by nanotubes, nanorods, nanoflowers, nanowires, nanofibers, fullerenes, dendrimers, and quantum dots (Tripathi and Chung, 2019). The microbiogenically synthesized nanostructures are preferred for their affectivity, convenience in production, and environment-friendliness (Ghosh et al., 2021). The microbial synthesis of NPs possesses various types of advantages in comparison to the other methods that include synthesis of nanomaterials with definite morphology, size, and chemical compositions. First of all, the synthesis can be performed under relatively mild physicochemical conditions. The convenience in handling the microbial cells results in the easy scaling up of the process and the ability to bring about the tuning of the characteristics of the nanomaterials by manipulating various cultivation parameters of the cultivation process (Prasad et al., 2016).

## Bacteria-Mediated Synthesis of Nanomaterials

For the last few decades, bacterial cells have been used for the purpose of synthesizing various types of inorganic nanomaterials that include gold, silver, selenium, and silver NPs possessing diverse useful properties (Wang et al., 2010). It is the colloidal properties of the gold nanoparticles that determine their antioxidant nature.

Abinaya et al. (2019) synthesized selenium nanowires (Se NWs) using microbial exopolymer (MEP) from *Bacillus licheniformis*, which was found to be effective in the management of biofilms. The synthesis of AuNPs occurs via the ligands that are produced by the microbial species to prevent the formation of complexes (Reith et al., 2009). The cells of *Rhodococcus* sp. were used for the development of monodispersed AuNPs (Ahmad et al., 2003). It has been further observed that *Deinococcus radiodurans* was able to synthesize AuNPs in the presence of high radiation that resulted in the change of Au (III) to Au (I) and finally to Au (0) comprising various types of capping groups which help in stabilizing the AuNPs (Li et al., 2016). Different types of biochemical processes are responsible for the synthesis of NPs. The intracellular mechanism of metal bioreduction is accomplished via the interactions of intracellular enzymes and positively charged groups that help in the gripping of metallic ions from the medium, causing subsequent reduction inside the cell

$${\text{H}}_{2}\to 2{\text{H}}^{+}+2{\text{e}}^{-}.$$

Transferring electrons to metal ions reduces them to metals in nanodimensions. For example,

$${\text{AuCl}}_{4}^{-}+3{\text{e}}^{-}\to \text{Au}+4{\text{Cl}}^{-}.$$

MNPs are thus formed on the surface of the cytoplasmic cell membrane due to the bioreduction of the metal ions by enzymes present on the cytoplasmic membrane and within the cytoplasm. In some cases, nucleation of MNPs was found to occur on the cell surface via enzymes and sugars in the cell wall, and later, metal nuclei were transported into the cell where they aggregated to larger sized particles. The process is initiated by the transfer of electrons from NADH by extracellular enzyme NADH reductase (Iravani, 2014).

In addition to the nitrate reductase enzymes, the carbonyl groups, such as –NH_2_, –OH, –SH, and –COOH, of some proteins and enzymes could stabilize the MNPs by binding to the NP surfaces by providing binding sites for metal ions, followed by the reduction of the metal ions outside the cells on the cell wall or in the periplasmic space. Bacteriogenic NPs can also be synthesized by metabolites of bacteria (Fang et al., 2019).

In the intracellular mechanism of metal bioreduction, interactions of intracellular enzymes and positively charged groups help in the gripping of metallic ions from the medium and the subsequent reduction inside the cell (Ovais et al., 2018).A number of physicochemical processes like complexation, nucleation, biosorption, stabilization, and growth are involved in the mechanism of nanoparticle synthesis. Various biomolecules that are associated with the bacterial cells like carbohydrates and proteins help in the stabilization of the NPs. It has been further observed that some groups of bacterial species like *Gluconacetobacter* help in the synthesis of nanocellulose. In comparison to nanofibrillated cellulose and nanocrystalline cellulose, bacterial nanocellulose possesses high purity, crystallinity, and large mechanical strength (Golmohammadi et al., 2017). The development of nanocellulose, a type of nanobiomaterial, has immense importance due to its biomedical applications (Morales-Narváez et al., 2015; Pourreza et al., 2015). The EPSs of the bacterial species comprise various functional groups that play an important role in the synthesis and the stabilization of the nanoparticles (Table 1) (Emam and Ahmed, 2016). The mucoadhesion properties of the EPSs result in the synthesis of NPs, thus resulting in the development of low surface energy, neutrality, and decrease in the low specificity recognition of the receptor capping, thereby making the NPs serve a wider applicability (Kanmani and Lim, 2013). The nanowires of bacterial origin are the groups of conductive proteinaceous pilus-like structures that are usually involved in the mechanism of electron transport by the involvement of the anaerobic dissimilatory metal-reducing groups of bacteria like *Shewanella* and *Geobacter* (Simonte et al., 2017) and aerobic bacterial species like *P. aeruginosa* (Simonte et al., 2017). Various types of bacterial species like *Acetobacter xylinum*, *Pseudomonas aeruginosa*, *Escherichia coli*, and many more can be used for the purpose of synthesizing PtNPs possessing a high potency of antibacterial and antibiofilm activities (Bloch et al., 2021).

**TABLE 1:** Properties of bacterial EPS aided synthesized nanoparticles used in nanomaterials.

Nanoparticle	EPS/component of EPS	Size	Morphology	Reference
AgNPs	EPS of *Lactobacillus casei*	0.2–10 nm	Spherical or rectangular in shape	Adebayo-Tayo and Popoola (2017)
ZnO NPs	EPS of *Bacillus licheniformis*	100 nm	Hexagonal in its dimensions	Abinaya et al. (2018)
FeO NPs	*Bacillus subtilis*	106 nm	Spherical in shape	Vignesh et al. (2015)
AgNPs	*Bradyrhizobium japonicum*	5–50 nm	Oval or rod-like in shape	Rasulov et al. (2016)
Au NPs	*Bacillus megaterium*	10 nms	Spherical in shape	Sathiyanarayanan et al. (2014)
Ag NPs	*Leuconostoc lactis*	35 nm	Spherical in shape	Saravanan et al. (2017)
Ag NPs	*Lactobacillus fermentum*	10 nm	Spherical or rectangular in shape	Adebayo-Tayo and Popoola (2017)
AuNPs and AgNPs	EPS from *Lactobacillus plantarum*	12–20 nm	Ellipsoidal or spherical in shape	Pradeepa et al. (2016)
AgNPs	EPS from *Lactobacillus brevis*	18 nm	Spherical in shape	Gomaa (2016)
AgNPs	EPS from *Lactobacillus rhamnosus*	10 nm	Hexagonal, triangular, and spherical in shape	Kanmani and Lim (2013)
AgNPs	Xanthan gum	5–40 nm	Spherical in shape	Xu et al. (2014)
AuNPs		15–20 nm	Spherical in shape	Pooja et al. (2014)
Pd-NPs		10 nm	Spherical in shape	Santoshi kumari et al. (2015)
Pd/FE-NPs		10–20 nm	Spherical in shape	Fan et al. (2013)
AuNPs	Dextran	13 nm	Spherical in shape	Medhat et al. (2017)
Zn-NPs	Curdlan	58 nm	Spherical in shape	Yan et al. (2016)

## Fungi-Associated Nanomaterial Synthesis

In recent times, fungi have been considered to be an important point of focus for synthesizing various types of nanomaterials and thus the development of the term myco-nanotechnology. Yeasts are found to be one of the most important types of fungi that play a significant role in synthesizing nanomaterials (Hulkoti and Taranath, 2014). Studies have shown the production of various water-soluble, biocompatible calcium telluride quantum dots by *Saccharomyces cerevisiae* having excellent physical characteristics. Such properties include flexibility of size under the influence of change of temperature and culture time and ability of photoluminescence, and these made them useful for various types of bio-labeling applications (Luo et al., 2014). *S. cerevisiae* also possesses the ability of synthesizing various types of Au–Ag alloy NPs that can be used for the purpose of various electrochemical sensor fabrications (Zheng et al., 2010). Fungi, as a whole, are considered to be one of the predominant sources for synthesizing nanomaterials due to their higher tolerance toward metals, higher ability of metal uptake and metal-binding capabilities, convenient way of culturing, and higher rates of synthesis of extracellular reductase enzymes (Syed et al., 2013) and other types of secondary metabolites (Dhillon et al., 2012). The fungal biomolecules help in the synthesis and stabilization of NPs (Syed et al., 2013).

## Microalgae-Associated Synthesis of Nanomaterials

The use of microalgae that are groups of photosynthetic microbial cells in the synthesis of nanomaterials has gained a lot of importance in the field of nanotechnology (Dahoumane et al., 2016). Synthesis of NPs using algal cells takes place by the accumulation of cations within the matrix of the cell, thereby bringing about reduction (Dahoumane et al., 2017). The mechanism of biosynthesis involves exposure of the salt to cell cultures or the biomass of algae, thereby synthesizing the NPs. In algal organisms like seaweeds, the reduction of the metallic salt is achieved by the biomolecules associated with the cell wall, thereby resulting in the synthesis of NPs.

## Mechanism of Biogenic Synthesis of Nanoparticles for Nanomaterials

A number of biogenically synthesized nanoparticles are reported from various bacterial, fungal, and algal species, which are found to have varied structure, size, and shape and are generally extracellularly synthesized. Later, they may be conjugated to other compounds to form nanomaterials (Table 2).

**TABLE 2:** Biogenically synthesized conjugated nanomaterials.

Type of nanomaterials	Microbial cell associated with the synthesis	Conditions required for synthesis	Characterization of the nanomaterials	Biosynthetic pathways	Reference
PbS NPs	*Aspergillus flavus*	0.5 mM of lead acetate with 6.4 mM of sodium sulfide along with the growth in potato dextrose agar at a temperature of 30°C for a period of 120 h and 150 rpm	Cubic crystalline structure, 35–100 nm	Extracellular synthesis	Priyanka et al. (2017)
ZnS: Gd NPs and ZnS	*Aspergillus flavus*	Fungal cells were grown in potato dextrose agar at 28°C for a period of 115 rpm. Along with the biomass, 3 mM of ZnSO_4_ was added at 27°C and 200 rpm. For the synthesis of ZnS:Gd NPs, 0.3 mM Gd(NO_3_)_3_ was added for a period of 96 h	Nanocrystalline structure, spherical structure, and 12–24 nm. ZnS: Gd NPs—10–18 nm	Extracellular synthesis	Uddandarao et al. (2019)
		ZnS:Gd nanoparticle 0.3 m			
Chitosan NPs	*Trichoderma harzianum*	Filtered biomass of the fungi that was grown in potato dextrose agar for a period of 72 h at 28°C at 180 rpm, followed by the addition	Spherical and amorphous, 98.8 nm	Synthesized extracellularly by enzymes	Saravanakumar et al. (2018)
AuNPs	*Penicillium brevicompactum*	Fungi were grown for a period of 72 h within potato dextrose broth at 30°C at 200 rpm. The filtered biomass was mixed in Milli Q sterile water and agitated at 30°C for a period of 72 h at 200 rpm. The supernatant was then mixed with HAuCl_4_ at a concentration of 1 mm at a temperature of 30°C in the dark	Hexagonal and triangular in shape. 25–60 nm	The NPs are synthesized extracellularly. Electrostatic interactions are responsible for the entrapment of ions with the fungal cell wall. The organic reagents that are present within the media are specifically used as reducing agents	Mishra et al. (2011)
AgNPs	*Fusarium oxysporum*	The fungi was grown in potato dextrose agar for a period of 5 days, followed by mixing the filtered biomass with 1 mm silver nitrate at 28°C for a period of 120 h in the dark	Face-centered cubic crystal, 5–13 nm	The reductase enzyme helps in the synthesis of the NPs	Husseiny et al. (2015)
AgNPs	*Humicola* sp.	The fungi were cultured in MGYP media at pH 9 and shaken at 200 rpm for a period of 50°C. This was followed by the addition of the mycelial mass with 1 mM AgNO_3_, which was shaken at 200 rpm, at a temperature of 50°C for a period of 96 h	Face-centered cubic crystal, spherical, and 5–13 nm	The biomolecules produced by the fungi helps in the extracellular synthesis of NPs	Syed et al. (2013)
TeNPs	*Aspergillus welwitschiae*	The fungi was grown in Czapek’s medium within a pH range of 7.3 at 30°C for a period of 5 days to which 2 mmol of K_2_TeO_3_ was added	Oval and spherical in shape, 60–80 nm		Abo Elsoud et al. (2018)
CdTe QDs	*Saccharomyces cerevisiae*	The fungi were grown under anaerobic conditions within Czapek’s medium for a period of 2 days. The cell aliquot stored at 5°C was added with 3 mM CdCl_2_ along with 0.8 mm Na_2_TeO_3_, 1.5 mm CH_3_SO_3_H, and 2.6 mm NaBH_4_, followed by rotation at 500 rpm	Cubic crystal, 2.6–3.0 nm	Extracellular synthesis of NPs	Luo et al. (2014)
Magnetosome chains	*Magnetospirillum gryphiswaldense*	Organisms that were grown micro-anaerobically were mixed with 50 µM of Fe(III)citrate	—	Genetic modification resulting in the enhancement of click beetle luciferase (CBR), thereby increasing the production of NPs	Roda et al. (2013)
γ-Fe_2_O_3_ magnetosome chains and individual γ-Fe_2_O_3_ magnetosomes	*Magnetospirillum magneticum*	The organism was grown micro-anaerobically	150–300 nm	The synthesis of the NPs occurs by the venous proteins that occur by genetic modifications and expression of RGD	Alphandéry et al. (2011)
Nanocomposites formed by bacterial nanocellulose with AgNPs AuNPs and CdSe and ZnS quantum dots that remain functionalized in the presence of biotinylated antibodies	*Acetobacter xylinum*	The synthesis of the NPs was performed within the static	45 ± 10 nm	Various types of extracellular and intracellular enzymes like glucokinase, phosphoglucomutase, pyrophosphgorylase, UDPG, and cellulose synthase	Morales-Narváez et al. (2015)
Bacterial nanocellulose fibrils	*Acetobacter xylinum*	Static culture enriched with polysaccharides	2–100 nms	Various types of extracellular and intracellular enzymes like glucokinase, phosphoglucomutase, pyrophosphorylase, UDPG, and cellulose synthase	Shao et al. (2015)
CdTe QDs	*Escherichia coli*	The bacterial cells were grown in Luria Bertani broth along with 3 mM CdCl_2,_ 0.8 mm Na_2_TeO_3_, 6 mM Na_3_C_6_H_5_O_7_, 26 mM NaBH_4_, and 8 mM C_4_H_6_O_4_S at 37°C for 24 h at 200 rpm	Cubic structure, size 2–3 nm	Produced extracellularly. Specifically, it is a protein-associated nuclear process	Bao et al. (2010)
Ag NPs	*Bacillus licheniformis*	Bacterial biomass was mixed with 1 mm AgNO_3_ at a temperature of 37°C	40–50 nm		Kalishwaralal et al. (2009)
Ag NPs	*Shewanella oneidensis*	Bacterial biomass was mixed with 1 mm AgNO_3_ at a temperature of 37°C	Spherical and crystalline in shape, 2–11 nm	Extracellular synthesis associated with NADH-dependent reductases	Suresh et al. (2010)
AuNPs and AgNPs	*Brevibacterium casei*	Bacterial biomass was mixed with 0.001 M AgNO_3_ and 0.001 HAuCl_4_ at a temperature of 37°C	10–50 nm whereas AuNPs are 0–50 nm	It allows intracellular synthesis of NPs which is an NADH-dependent nitrate reductase for AgNPs and α-NADPH–dependent sulfite reductase for AuNPs	Kalishwaralal et al. (2010)
Au NPs	*Nocardiopsis* sp.	Cell-free supernatant was added with 1 mm HAuCl_4_	Spherical in shape, 12 ± 5 nm	It is an extracellular mechanism of synthesis where enzyme-based shuttle-based enzymatic reduction of ionic Au^3+^ to Au^0^ occurs	Suresh et al. (2011)
Se NPs	*Pantoea agglomerans*	Overnight-grown culture within trypic soy broth added to 1 mm Na_2_SeO_3_ at a temperature of 25°C for a period of 24 h	Amorphous and spherical shaped, 100 nm	Se (III) is reduced to Se(0) by the mechanism of intracellular reduction	Torres et al. (2012)
Se NPs	*Streptomyces minutiscleroticus*	The bacterial biomass was grown for a period of 120 h. To that, 1 mm Na_2_SeO_3_ was added, followed by stirring at 200 rpm	Spherical and crystalline in shape, 10–250 nm	Extracellular synthesis of NPs	Ramya et al. (2015)
Polycrystalline AgNPs	*Amphora*	The growth is achieved within F/2 media within filtered sterile brackish water maintained at pH 8.2 at a temperature of 30°C for a period of 16.8 h at a rotation of 120 rpm followed by addition of 2 mm Silver nitrate	20–25 nm	This involves the process of extracellular synthesis of NPs where fucoxanthin is involved	Jena et al. (2015)
Au NPs with biogenic silica	Fossil diatoms	NA	10–30 µm	NA	Panwar and Dutta (2019)
Biogenic silica	*Thalassiosira weissflogii*	The growth of the organism was achieved in silicate-rich sea water media at a temperature of 18–20°C for a period of 12:12 light and dark cycles	NA	Natural process of biomineralization	Lo Presti et al. (2018)
Streptomycin loaded within biogenic silica	*Coscinodiscus concinnus*	NA	220 µm	Natural process of biomineralization	Gnanamoorthy et al. (2014)
AuNPs	*Tetraselmis kochinensis*	The organism was grown within Guillard’s Marine Enrichment media at a temperature of 28°C for a period of 15 days under light conditions, followed by the addition of the supernatant with 1 mM HAuCl_4_ at a rotation of 200 rpm and a temperature of 28–29°C	5–35 nm	Intracellular synthesis of NPs by means of active compounds that are associated with the cell wall and the cytoplasm	Senapati et al. (2012)

## Role of Nanomaterials in Disintegration of Biofilm

About 80% of the microbial infections that occur within the body are associated with biofilms, and hence, it has resulted in a serious concern among healthcare personnel. Various studies showed that nosocomial organisms like *S. epidermidis*, *S. aureus*, and *P. aeruginosa* possess the ability to form biofilms very rapidly on the surfaces of medical devices. It has been observed that a biofilm is constituted of three layers: the initial layer remains adhered to the surface of the biomaterial, the next layer comprises the microcolonies, and the outer layer comprises planktonic organisms that remain free on the outer surface and possess the ability to get dispersed to the surroundings (Kunin, 1989; Costerton et al., 1999; Reid, 1999; Bernier et al., 2003)

## Extracellular Polymeric Substance of Biofilm Matrix

In biofilms, the consortia of sessile microbial colonies remain adhered to a biotic or abiotic surface within the self-secreted extracellular polymeric substance (EPS), which comprises exopolysaccharides, proteins, lipids, nucleic acid, and various other types of biomolecules (Flemming et al., 2016; Lahiri et al., 2021c). EPS helps in the bacterial adhesion upon the surface and acts as cementing material between the cells, allowing them to remain in very close association, thereby allowing interactions between the cells (Dragoš and Kovács, 2017; Koo et al., 2017; Lahiri et al., 2021a).Various types of polymers that are of secondary origin like colloids and humic substances also remain embedded within the biofilm.

## Mechanisms Associated With the Disruption of Biofilm

**FIGURE 1:**
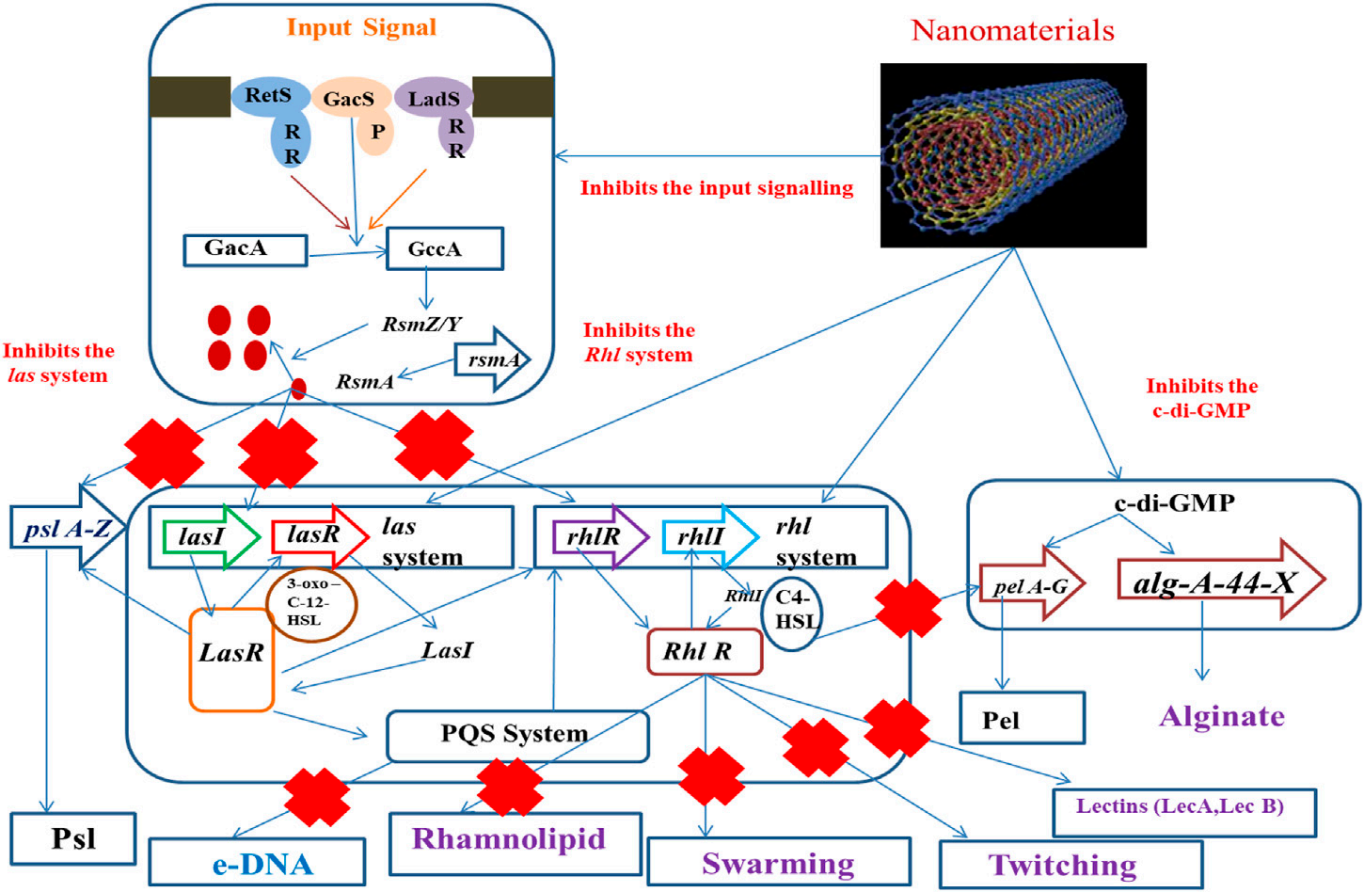
Action of nanostructures at different points of the biofilm formation event.

Now, most biofilm eradication approaches involve the development of antibiofilm agents, aimed at preventing the early stages of biofilm construction or acting as biofilm dispersal agents, intended to cause disruption of the mature biofilm. They may follow any of the potential ways like checking of quorum sensing, destruction of eDNA, and affecting swarming and twitching (Figure 1) or can directly damage the biofilm-forming cell.

## Nanomaterials With Direct Antibiofilm Property

Nanomaterials formed of microbiogenic nanoparticles like nanosilver or nanogold particles efficiently block the active sites and thus hinder the mechanism of quorum sensing (Lahiri et al., 2021b). They can inhibit the metabolic events for the EPS production, thereby hampering the formation of biofilm (Samanta et al., 2017).

The interaction between NPs and biofilm can be accomplished through the allocation of nanoparticles of the nanomaterials at the vicinity of the biofilm matrix, followed by their attachment. NPs, due to electrostatic interaction, can now bring about mechanical damage to the bacterial cell or can develop oxidative stress followed by the production of reactive oxygen species (ROS) and, as a result of metallic cation release, can interrupt the normal structure and functions of proteins (Shkodenko et al., 2020).

## Alteration in Biofilm-Forming Signaling Pathways

Alteration in the signaling cascade involves the prevention of the production of EPS that provides an alternate mechanism for preventing the adhesion of bacteria, thus hindering the formation of biofilms. Thus, it forms a very important target for the next-generation therapeutics (Sintim et al., 2010). In recent times, various studies have been conducted by the utilization of anti-QS agents that prevent the adherence of bacterial species by surface modifications (Kratochvil et al., 2015). Surface immobilization by the use of QS-inhibiting agents also prevents the development of biofilms (Brackman et al., 2016; Kim et al., 2017)

## Strategies of Development of Antibiofilm Agent Targeting Extracellular DNA

**FIGURE 2:**
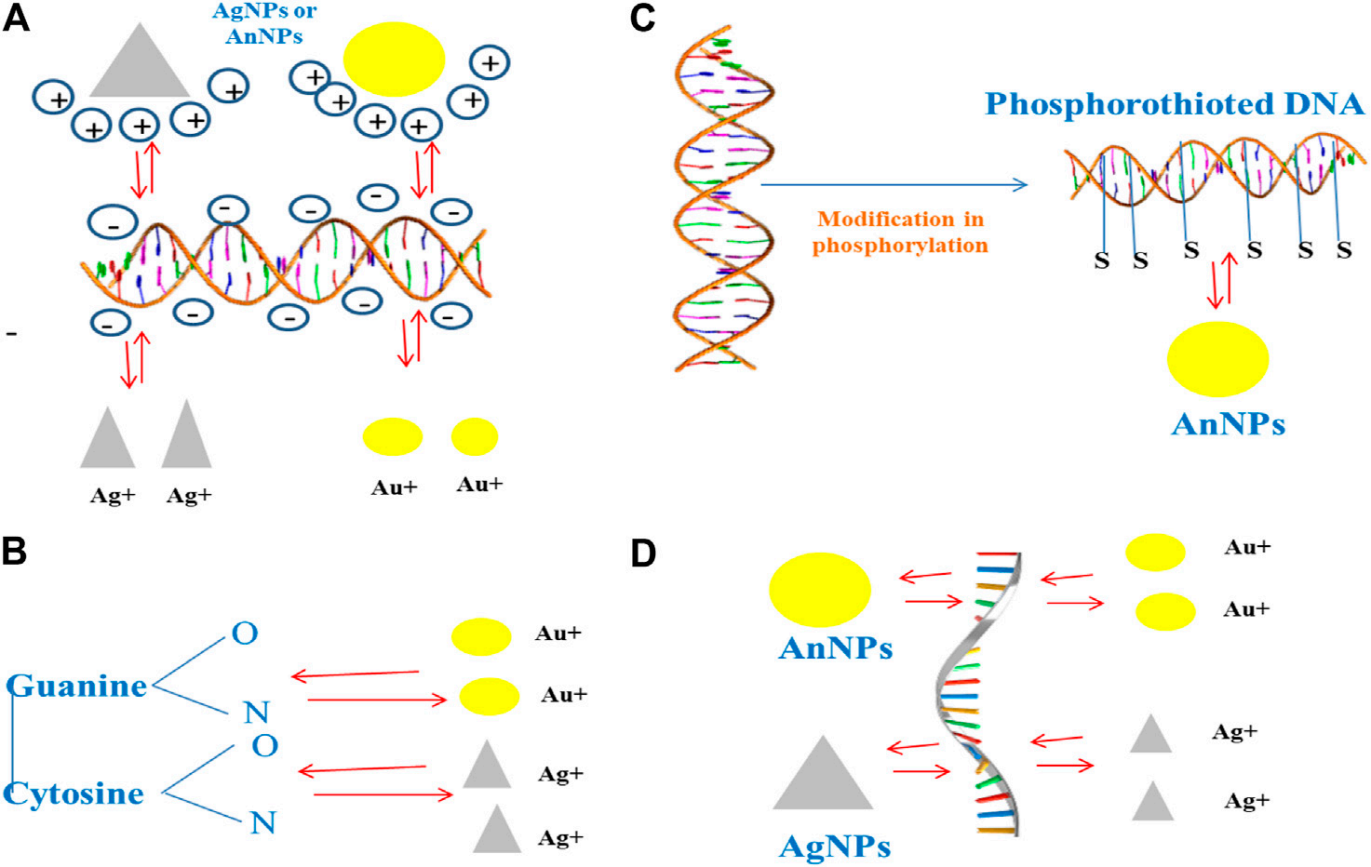
Interaction of NPs with the eDNA present within the biofilm.

Extracellular DNA (eDNA) forms an important structural component in stabilizing the biofilm architecture and develops resistance to drugs. It has been observed that substances like amphiphilic cargo and enzymes bring about the cleavage of eDNA, thereby bringing about effective degradation of biofilm (Swartjes et al., 2013). eDNA is polyanionic in nature and shows interactions with AuNPs and AgNPs, which are the positively charged molecules. Studies have shown that AuNPs exhibit covalent as well as noncovalent interactions with the backbone of polyanionic eDNA (Carnerero et al., 2017). The gold and silver ions that come from the respective NPs interact with the nitrogen and oxygen atoms that are associated with the nitrogen bases that are present within the DNA background with the Van der Waals and hydrophobic forces, but the electrostatic forces are dominant over the Van der Waals force (Koo et al., 2015; Jiang and Ran, 2018; Radzig et al., 2019). The degradation of DNA was achieved by the interaction along with the binding of gold ions, and this was an important reason to bring about the damage of DNA in place of the ROSs that are being produced by AuNPs. It has been further observed that damage caused by ROS-mediated oxidation induces the repair mechanism of DNA within bacteria. However, the mutant group bacterial strains showed impaired DNA repair mechanisms and are more vulnerable with respect to the wild-type strains to the gold ions (Radzig et al., 2019). Studies have showed that phosphorothionation brings about modification within bacterial DNA, protecting it from various types of unfavorable environmental conditions (Howard et al., 2013). AuNPs can easily react with these DNA and bring about the change in their chemistry, thus resulting in disintegration of the DNA (Figure 2).

## Mechanism of Extracellular Polymeric Substance Disruption With Biogenic Nanoparticles

The extracellular matrix provides strength to the indwelling sessile microbial species within the biofilm by virtue of various biomacromolecules known as EPSs, thereby contributing toward shortened antimicrobial susceptibility. So far, EPS targeting for biofilm control has remained underexploited due to lower penetration capabilities of various antibiofilm agents such as antibiotics, biofilm degrading enzymes, and bioactive compounds. Nanoparticles (NPs) have emerged either as EPS matrix disruptors or as carriers of EPS matrix disruptors, and several approaches have recently been proposed (Fulaz et al., 2019). NPs have also been observed to interfere with the cell–cell communication signaling cascade, thus acting as quorum sensing inhibitors (Naik and Kowshik, 2014; Miller et al., 2015; Singh et al., 2015; Al-Shabib et al., 2016; Srinivasan et al., 2017).

EPSs (polysaccharide skeleton, proteins, and DNA) or bacterial cells are, in general, negatively charged, hence providing an efficient way of interaction with positively charged NPs (Flemming et al., 2000; Regiel-Futyra et al., 2017). It has been observed that cationic NPs can penetrate and diffuse well within the matrix as compared to neutral or anionic NPs (Li et al., 2015). It has also been observed that hydrophilic NPs have poorer localization effects within the bacterial cells than hydrophobic NPs due to the formation of stable EPS-hydrophobic components with the NPs (Mitzel et al., 2016). EPS comprises proteins (TasA, TapB, BslA, SipW, CdrA, and lectins), eDNA, and polysaccharides (Pel, Psl, PIA, alginate, and cellulose) that are responsible for adhesion, water retention, aggregation, cohesion, redox reactions, and enzymatic activity and provide structural integrity and a protective barrier to the biofilms. The most important NP–biofilm interactions involve electrostatic, hydrophobic, and steric forces. Electrostatic interactions are mainly responsible for the initial adhesion to surfaces (biofilm matrix or bacterial cells) (Flemming and Wingender, 2010; Habimana et al., 2014). Hydrophobic interactions play a major role in the biofilm formation and its regulation (Renner and Weibel, 2011; Flemming et al., 2016). Steric interactions are needed for colloidal stabilization of the NPs, preventing their self-aggregation (Huangfu et al., 2019).

In a study conducted by Cremonini et al. (2016), selenium nanoparticles (SeNPs) of bacterial origin were reported to stop biofilm formation and disassemble mature glycocalyx of *P. aeruginosa* and *Candida* spp*.* The *Stenotrophomonas maltophilia* [Sm-SeNPs(-)] and *Bacillus mycoides* [Bm-SeNPs(+)] had stronger antimicrobial effects than synthetic selenium nanoparticles (Ch-SeNPs) (Cremonini et al., 2016). Thus, biogenic SeNPs appear to be reliable candidates for safe medical applications alone. In another work, biogenic AgNPs synthesized using *Desertifilum* sp. (D-SNPs) were able to inhibit biofilms of MRSA, resulting in imbalance in CAT, GSH, GPx, and ATPase levels and subsequently forming apoptotic bodies and causing cell wall damage in addition to denaturation of MRSA cellular proteins and genotoxicity (Hamida et al., 2020).

**FIGURE 3:**
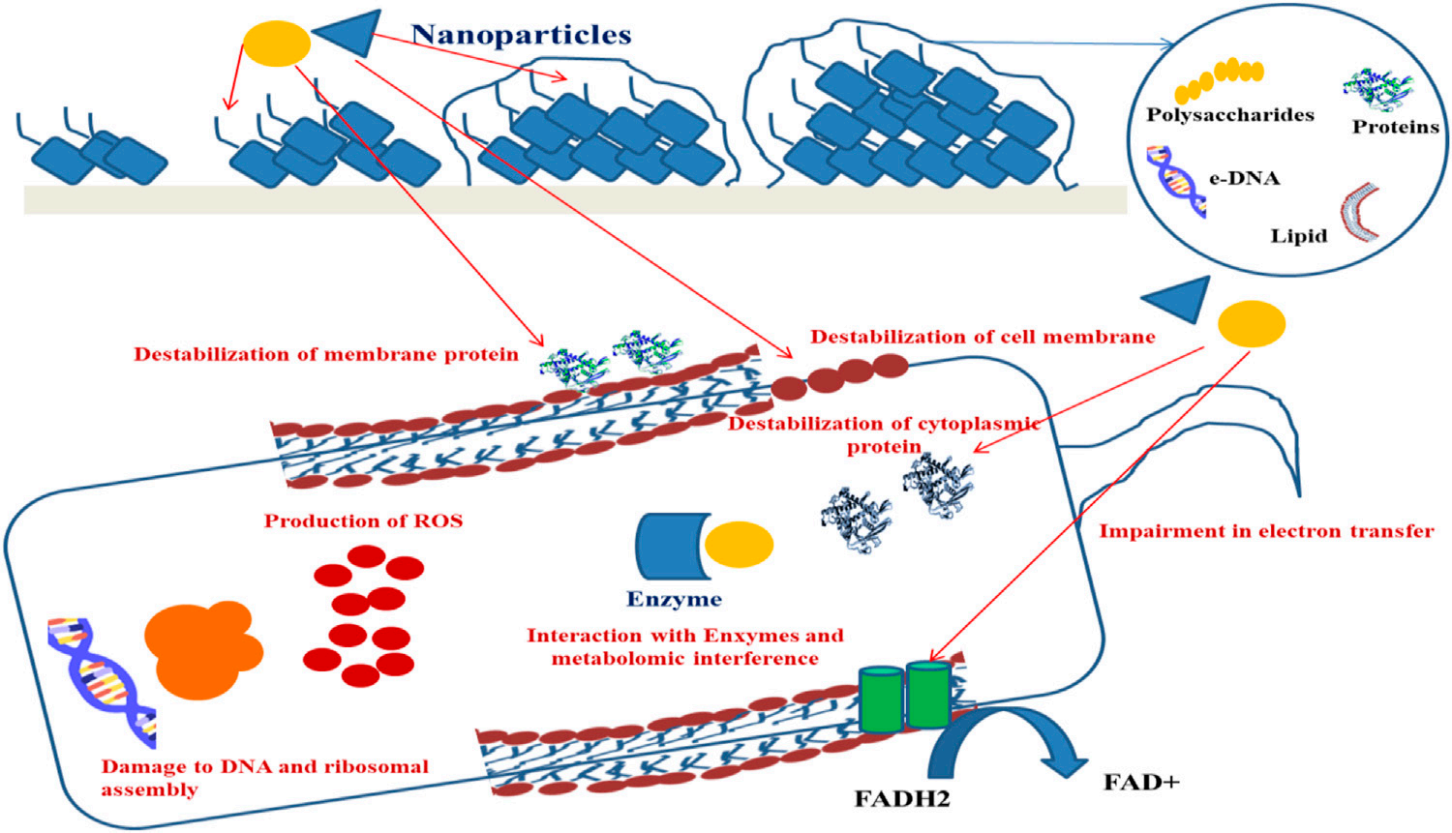
Mechanism of NPs bringing about inhibition of biofilm.

Owing to the high surface-to-volume ratio, NPs possess an efficient transport phenomenon within the biofilm matrix. The size of NPs controls the initial penetration within the matrix, and the NP surface properties, namely, charge and functional groups control the mode of interaction with the matrix components. The presence of organic molecules (proteins, lipids, nucleic acids, carbohydrates, metabolites, etc.) within the biofilm matrix has been reported to be responsible for the formation of a biomolecular corona-like coating on the surface of NPs due to the phenomenon of adsorption on the NP surface (Mu et al., 2014; Docter et al., 2015; Ikuma et al., 2015; Ke et al., 2017; Stan et al., 2018). The physicochemical properties of NPs involve characteristics like size, shape, hydrophobicity, surface charge, curvature, and functionalization that are responsible for the altered interaction between NPs and biofilm matrices or microbial cells (Canesi and Corsi, 2016; Mi et al., 2018). For example, adsorption of NPs on the microbial cell surface has been observed to cause cellular membrane puncture, along with generation of reactive oxygen species (ROS), inhibiting mitochondrial activity, protein, and DNA synthesis (Hajipour et al., 2012; Joo and Aggarwal, 2018). Copper NPs synthesized by *P. aeruginosa* were found to increase the velocity of wound healing (Tiwari et al., 2014), whereas silver NPs from *P. chrysogenum* were found to be effective against the biofilm-producing bacteria *S. aureus*, *P. aeruginosa, E. coli*, and *B.cereus* (Akila et al., 2014). Nanomaterials can be successfully applied to remove or check device-associated biofilm formation (Table 3, Figure 3).

**TABLE 3:** Examples of effective application of nanomaterials against device-associated biofilm

Antibiofilm activity of nanomaterials	Antibiofilm implants on device	Mechanism	Reference
Zinc-associated copper oxide nanocomposite (Zn-CuO)	Contact lenses	Zn-CuO nanocoating being present upon the surface of the lenses prevents the development of biofilm upon their surface	Tuby et al. (2016)
Silica NPs	Contact lenses	It possesses brush coatings on the polypropylene cases that inhibit the development of biofilm in comparison to the uncoated polypropylene. It also prevents the spreading of microbial colonies upon the surface of the lenses	Qu et al. (2013)
Silicone NPs	Used in breast implants	It helps in the reduction of immune responses that are generated by peripheral mononuclear blood cells and can be effectively be used in preventing the development of biofilm	Nair et al. (2012)
NPs releasing nitric oxides	Catheters	It plays an effective role in preventing the development of biofilm. It especially prevents the biofilm of *S. aureus* by inhibiting the EPS being produced by them	Nair et al. (2012)
Ag-Ti nanocomposites	Used within face masks	It prevents the development of biofilm by *S. aureus* and *E. coli*	Li et al. (2006)
Silver conjugated NPs	Used in prosthetic heart valves	It prevents the development of biofilm by interfering with the sessile colonies	Angelina et al. (2017)
ZnO NPs along with titanium implants	Used in various types of orthopedic implants	The Ti being present within the ZnO–Ti nanocomposites helps in promoting adhesion of mammalian cells and thereby inhibits the bacterial cell adhesion	Elizabeth et al. (2014)
Titania nanostructure coated with AgNPs	Used in oral implants and endodontic filing	It helps in the killing of the planktonic cells and also prevents the development of the biofilm	Zhao et al. (2011)

## Conclusion

The biofilm matrix, also sometimes known as “the dark matter,” is a complex material which creates a barrier shielding the indwelling cells from antimicrobial therapy, immune responses, and environmental challenges and hence prevents eradication strategies. Due to the outstanding challenges presented by the biofilm matrix, a multidisciplinary approach is needed to tackle this problem. Nanotechnology is a plausible solution for antimicrobial and delivery system methodologies for enhanced penetration and targeted delivery of antimicrobials within the biofilm matrix. EPS-targeting strategies involve matrix disruption and enhancing the susceptibility of the biofilm toward antimicrobial therapy.

One of the ways for the synthesis of biogenic NPs involves microbial cells as a reducing, stabilizing, and capping agent in an eco-friendly, sustainable, nontoxic, and inexpensive way. Many researchers have studied the role of bacteria (both Gram-positive and Gram-negative), fungi, or algae in the production of NPs. These methods have resulted in the replacement of various toxic physicochemical methods. However, a few of the questions such as alterations in EPS composition during different environmental/growth conditions, non-commercialization of NP-based antibiofilm technologies, ultimate fate of antibiofilm NPs *in vivo*, and release of NPs into the environment still remain to be answered. Future research work should highlight the complete biofilm eradication by focusing on both the EPS matrix and the microbial cells, minimizing toxicity and resistance development while enhancing the therapeutic effect with the help of nanostructures formed from microbial sources.
